# Steroids and Alzheimer’s Disease: Changes Associated with Pathology and Therapeutic Potential

**DOI:** 10.3390/ijms21134812

**Published:** 2020-07-07

**Authors:** Yvette Akwa

**Affiliations:** “Disease and Hormones of the Nervous System”, U1195 Inserm-Université Paris Saclay, 80 rue du Général Leclerc, 94276 Kremlin-Bicêtre, France; yvette.akwa@inserm.fr

**Keywords:** neurosteroids, neuroactive steroids, sex difference, amyloid-β, tau protein, neuroprotection, mitochondria, neuroinflammation, memory, TSPO

## Abstract

Alzheimer’s disease (AD) is a multifactorial age-related neurodegenerative disease that today has no effective treatment to prevent or slow its progression. Neuroactive steroids, including neurosteroids and sex steroids, have attracted attention as potential suitable candidates to alleviate AD pathology. Accumulating evidence shows that they exhibit pleiotropic neuroprotective properties that are relevant for AD. This review focuses on the relationship between selected neuroactive steroids and the main aspects of AD disease, pointing out contributions and gaps with reference to sex differences. We take into account the regulation of brain steroid concentrations associated with human AD pathology. Consideration is given to preclinical studies in AD models providing current knowledge on the neuroprotection offered by neuroactive (neuro)steroids on major AD pathogenic factors, such as amyloid-β (Aβ) and tau pathology, mitochondrial impairment, neuroinflammation, neurogenesis and memory loss. Stimulating endogenous steroid production opens a new steroid-based strategy to potentially overcome AD pathology. This article is part of a Special Issue entitled Steroids and the Nervous System.

## 1. Introduction

Alzheimer’s disease (AD) is the most common dementia of the elderly and remains one of today’s biggest public health challenges. The main pathological features observed in the AD brain are loss of synapses at early stages of the disease, senile plaques of amyloid-β (Aβ) peptides, neurofibrillary tangles (NFTs) containing hyper and abnormally phosphorylated tau proteins leading to progressive neuronal loss (cortical atrophy). One form of the disease is familial AD, a very rare pure autosomal dominant disease with early onset before 65 years, which is caused by mutations in amyloid precursor protein (APP), presenilin-1 (PS1) or presenilin-2 genes all connected to Aβ accumulation. The other form is sporadic AD or late-onset AD which accounts for the majority of AD cases and is caused by environmental factors and genetic predisposition [1]. Soluble Aβ oligomers impact synaptic plasticity early in the pathological process [2,3]. Progressive synaptic dysfunction then impairs episodic/recent memory followed by slow decline in other cognitive abilities, accompanied by neuropsychiatric symptoms including apathy, anxiety and depression [4,5]. A large body of evidence suggests that there is a long prodromal infraclinical phase during which pathological changes begin decades before plaques and tangles are formed [6].

Besides aging, which is considered the greatest risk factor of AD, apolipoprotein (ApoE) ε4 genotype and sex are critical AD risk factors [7,8]. Several neurobiological pathways also contribute to neurodegeneration and cognitive impairment, including mitochondrial dysfunction and oxidative stress, neuroinflammations that are pertinent targets of neuroactive neurosteroids.

At present, no medication exists for AD despite intensive research on neuropathology, symptoms and mechanisms. Symptomatic treatments have had either little or no effect, so new strategies mostly aim at reducing the overall burden within the AD brain. Among them, the exogenous administration of neuroactive steroids or the modulation of their endogenous production can potentially provide therapeutic benefits, particularly in the preclinical stage before the neurodegenerative disease process is established. Endogenous neuroactive steroids include steroids that are synthesized de novo in the central nervous system (CNS) as neurosteroids, hormonal steroids generated from endocrine glands and transported into the brain from circulation, and steroids synthesized in the brain from gonadal steroids. Significant alterations in their concentration and metabolism are observed in the blood, brain and cerebrospinal fluid (CSF) samples of AD patients. In addition, neuroactive steroids, independent of their central or peripheral origin, regulate a wide range of key physiological processes in the CNS by regulating gene expression, neuronal excitability and signaling. These data have provided the basis for their neuroprotective, neuroregenerative and neuropsychopharmacological effects that may be pertinent for AD treatment [9,10,11].

The aim of this review is to summarize the current research on the levels of steroids and biosynthetic enzymes in the AD brain and their relationship with critical pathogenic factors in various AD models, including human neuroblastoma cell lines, rats and transgenic mice developing Aβ or tau pathology. Emphasis is on steroid specificity and, when possible, on sex differences. The focus is on key neuroactive steroids, namely the neurosteroids pregnenolone (PREG) progesterone (PROG), alloprogregnanolone, dehydroepiandrosterone (DHEA), the sulfated steroids pregnenolone sulfate (PREGS) and dehydroepiandrosterone sulfate (DHEAS), as well as the sex-steroid testosterone and 17β-estradiol (E2).

## 2. Dysregulated Brain Steroidogenesis and Steroid Concentrations Associated with AD

### 2.1. Steroidogenesis in the Human Brain

**Figure 1:**
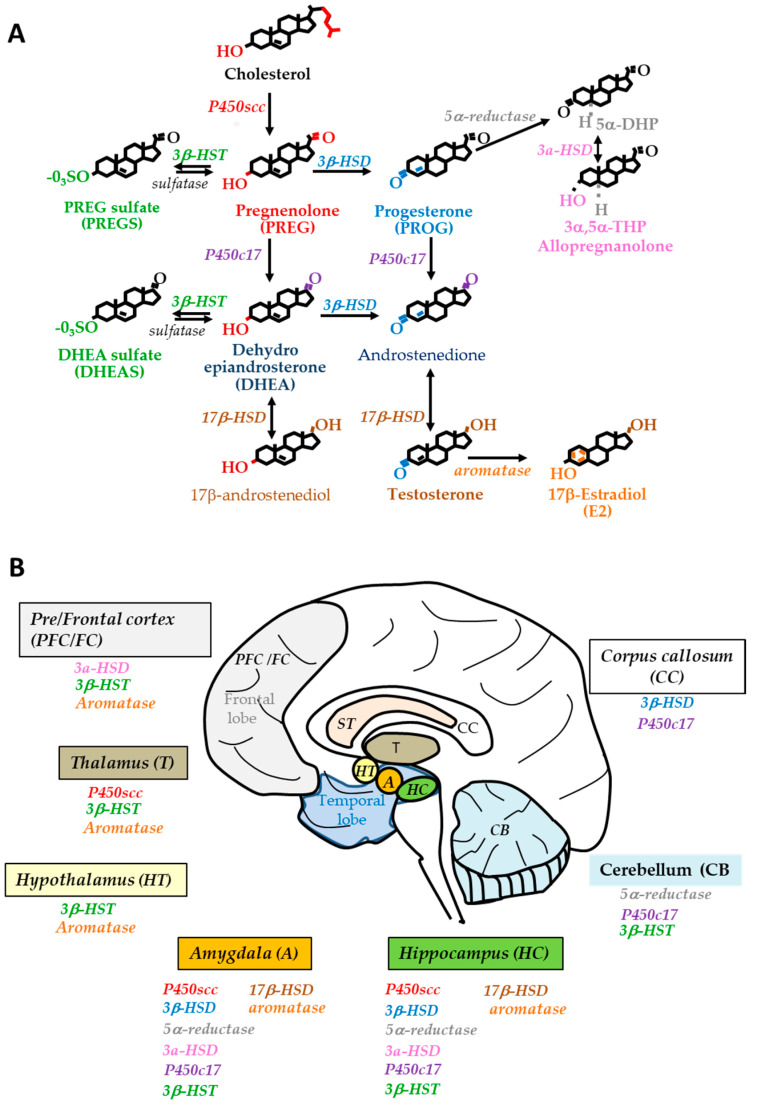
Neurosteroid and sex steroid biosynthetic pathway in the human brain. (**A**) The main steps of neuroactive steroid synthesis are shown. The initial and limiting step involves P450scc activity catalyzing the metabolism of cholesterol to pregnenolone (PREG), the precursor of all neurosteroids and sex steroids. Then, PREG is transformed to progesterone (PROG) and its 5α-reduced and 5α,3α-reduced metabolites (5α-DHP and allopregnanolone). These steps involve 3β-HSD and the 5α-reductase/3α-HSD complex enzymes. PREG and PROG are precursors of DHEA and androstenedione, respectively. PREGS and DHEAS are produced from PREG and DHEA by sulfotransferases and can in turn be desulfated by sulfatases. The so-called sex steroids are testosterone produced from androstenedione by 17β-HSD activity and E2 from testosterone aromatization. (**B**) Location of steroidogenic enzyme in the human brain regions. Enzyme mRNA, protein and activity are present, except for P450scc whose mRNA was only expressed. P450scc: P450side chain cleavage, 3β-HSD: 3β-hydroxysteroid dehydrogenase-Δ5 (Δ4 isomerase, 3α-HSD: hydrosteroid dehydrogenase, 3β-HST: 3β-hydroxysteroid sulfotransferase, 17β-HSD: 17β-hydroxysteroid dehydrogenase 5α-DHP: 5α-dihydroprogesterone; 3α,5α-THP: 3α,5α-tetrahydroprogesterone.

Since human brain steroidogenesis can differ from that of laboratory animals, investigating steroid and enzyme levels also requires samples from AD patients and age- and gender-matched nondemented controls. Moreover, blood steroid levels do not necessarily adequately reflect brain steroid concentrations and enzyme activity. All steroids are synthesized from cholesterol either de novo from acetate in the endoplasmic reticulum or imported from density lipoproteins derived from dietary sources. Steroidogenic pathways are well characterized in rodent brains [12]. The human brain also has the capacity to synthesize steroids. Evidence supports the existence of key steroidogenic enzyme mRNAs, protein or activity in brain samples. The biosynthesis and metabolism of neurosteroids and sex steroids in the human brain is depicted in Figure 1. The cytochrome P450side-chain cleavage (P450scc), encoded by the Cyp11A1 gene, is involved in the initial and rate-limiting step inside mitochondrial matrix that converts cholesterol to pregnenolone (PREG), the precursor of all steroids [13]. P450scc mRNA was found in several areas, i.e., the temporal and frontal neocortex, hippocampus, corpus callosum, thalamus, caudate nucleus and amygdala [14,15]. The corresponding protein was detected in the cerebellar white matter by immunochemistry [16]. The 3β-Hydroxysteroid dehydrogenase-Δ5→Δ4 isomerase (3β-HSD) is a membrane-bound enzyme located in the mitochondria and endoplasmic reticulum. It catalyzes the production of PROG from PREG and androstenedione from DHEA. In humans, type 1 and type 2 3β-HSD isoforms are characterized. Both 3β-HSD isoform mRNAs were present in the corpus callosum, hippocampus and amygdala, with type 2 at higher levels than type 1 [15]. PROG can act in its native form and/or after transformation into active metabolites. In the CNS, the 5α-reductase converts PROG to 5α-dihydroprogesterone (5α-DHP) which is further reduced by the 3α-hydroxysteroid dehydrogenase (3α-HSD) to the potent γ-aminobutyric acid type A (GABA_A_) receptor-acting neurosteroid 3α,5α-tetrahydroprogesterone (3α,5α-THP) also named allopregnanolone. Two forms of 5α-reductase exist, with type 1 being the major isoform widely distributed in the human brain. Type 1 5α-reductase mRNA and activity are located in the temporal neocortex, subcortical white matter and hippocampus [14,17]. The 3α-HSD belongs to the aldo-keto reductase (AKR) superfamily with AKR_1_C_1_, AKR_1_C_2_ and AKR_1_C_3_ isoenzymes expressed in the human brain [18]. AKR_1_C_2_ (type 3 3α-HSD) metabolizes 5α-DHP to allopregnanolone while AKR_1_C_1_ metabolizes allopregnanolone to its 20α-reduced metabolite thus reducing neurosteroid concentration in the brain [19,20]. AKR_1_C_3_ (3α-HSD type 2) mRNA was characterized in the temporal neocortex and subcortical white matter [17]. Interestingly, mRNA expression of 3α-HSD was much higher than that of 5α-reductase, suggesting that 5α-reduction is the rate-limiting step in the synthesis of allopregnanolone. The neurosteroids PREG and PROG can also be converted by the cytochrome P450c17. This 17 α-hydroxylase/17, 20-lyase (CYP17) converts both steroids into DHEA and androstenedione, respectively. The level of P450c17 mRNA was higher in the corpus callosum and amygdala than the hippocampus and cerebellum [15]. With respect to sulfated steroids, human 3β-hydroxysteroid sulfotransferases (3β-HSTs) are present in the human brain and SULT2B1 is selective for the sulfonation of 3β-hydroxysteroids, such as PREG and DHEA [21,22]. Only SULT2B1b (not SULT2B1a) transcripts were detected throughout the human brain regions with the frontal/temporal lobes and thalamus expressing the highest levels [22].

The 17β-hydroxysteroid dehydrogenases (17β-HSD) and aromatase are essential in the end steps of neurosteroidogenesis. The 17β-HSD is involved in the interconversion of androstenedione to the strong androgenic testosterone, and DHEA to 17β-androstenediol. The expression of mRNAs encoding type 1, 3, 4, 5, 7, 8 and 10 isoforms of 17β-HSD was detected in the human temporal lobe [14,23]. The in vitro activity of 17β-HSD promoted the synthesis of testosterone in the cerebral cortex and subcortical white matter [24] and of 17β-androstenediol in several human brain regions, including the hippocampus, amygdala striatum and cerebellum [25]. 17β-HSD10, known to be involved in the inactivation of many endogenous steroids, was found to be highly expressed in the human hippocampus, hypothalamus and amygdala [26]. At last, the cytochrome P450 aromatase, encoded by Cyp19 gene, is another key enzyme for the aromatization of testosterone to E2. Aromatase mRNA expression was detected in the frontal cortex, hippocampus, subcortical white matter, thalamus and hypothalamus [14,27,28,29]. Aromatase activity was demonstrated in human frontal and temporal brain regions [30,31], hippocampus, pons, thalamus and hypothalamus [28,32]. Notably, 17 β-HSD plays a critical role in the bidirectional reactions between E2 and the weak estrogenic compound estrone E1, and the 17 β-HSD type 1 catalyzed the reduction of E1 to E2 [33].

To date, no sex-specific differences have been identified for all the enzymes cited above except for P450scc, for which mRNAs levels were higher in adult women than men, particularly in the temporal lobe, frontal lobe and hippocampus [34,35]. The steroid biosynthetic pathway and steroid enzyme location in the human brain are depicted in Figure 1.

### 2.2. Changes in Neurosteroid and Biosynthetic Enzyme Levels

**Figure 2:**
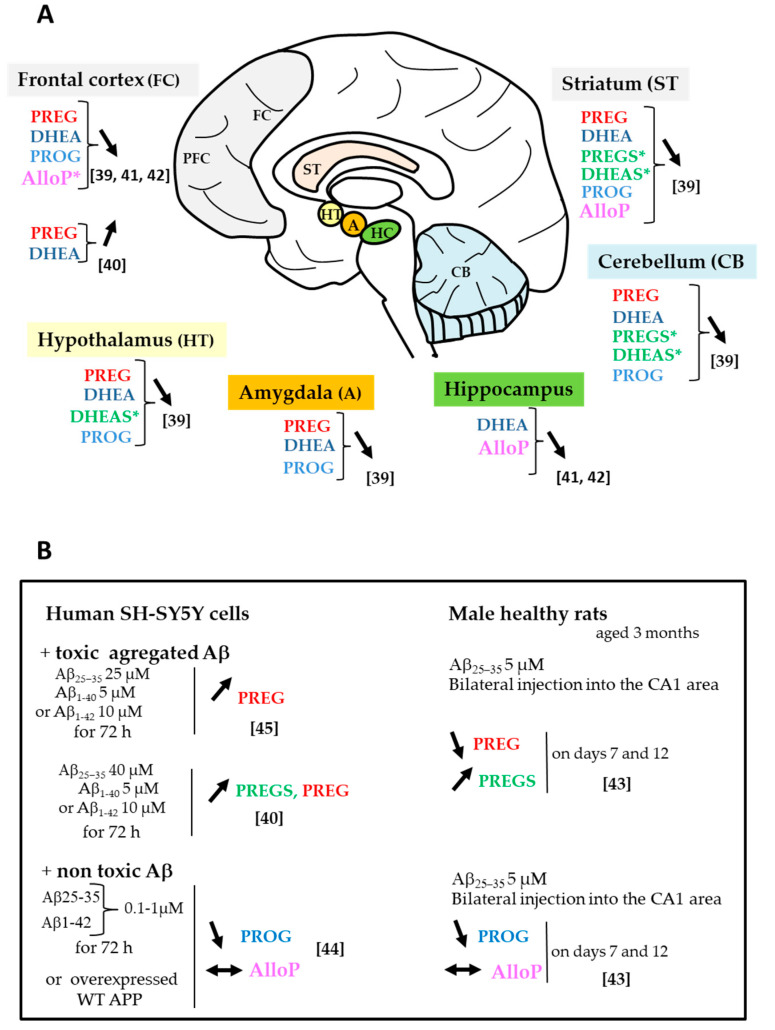
Change in endogenous neurosteroid levels. (**A**) In the AD brain. A general trend of lower levels of neuroactive steroids was observed in different brain regions of AD patients than nondemented controls (*significant decrease in steroid levels). Participants were women and men with mean age 86 ± 2 years [39], 77.3 ± 2 years [42] or only men with median age 83.0 years [41]. (**B**) Under in vitro and in vivo Aβ burden conditions. ↓: decrease, ↑: increase, ↔: no change vs. Aβ.

Increasing evidence suggests that dysregulation of steroid endogenous concentrations and their biosynthetic enzymes play a role in neurological diseases, including AD [36,37]. It is to be noted that brain steroid levels reflect not only hormonal production and metabolism from endocrine glands, but also the local neurosteroidogenesis. There is a very limited or no recent data addressing brain steroid levels in AD or control subjects. Therefore, we choose to cite the few initial studies that described substantial changes in neurosteroid levels in postmortem brain samples of AD patients as compared to cognitively intact nondemented subjects. Those investigations used reliable methodologies, coupling solid phase extraction, purification by high performance liquid chromatography and identification by gas chromatography-mass spectrometry (GC-MS) instead of radioimmunoassay. Indeed, the latter represents an important limitation in analyzing low concentrations of steroids in brain tissue samples, due mainly to the lack of specificity and availability of antibodies [38]. Numerous neurosteroids have been quantified in postmortem human brain specimens. Substantial changes in their levels in the AD brain (Figure 2A) suggest that disequilibrium in neurosteroid pathways have a role in AD pathogenesis. The regulation of steroid levels by Aβ burden in vitro and in vivo is described (Figure 2B).

PREGS and DHEAS were found to be significantly lower in aged AD patients than age-matched nondemented controls, especially in the striatum, cerebellum and hypothalamus, and negatively correlated with high levels of cortical Aβ and phosphorylated tau proteins [39]. These reduced PREGS and DHEAS levels suggested that the 3β-HST enzyme involved in their biosynthesis was reduced. However, Calan et al. 2016 [40] showed that toxic doses of Aβ significantly increased PREGS cellular levels in a time-dependent manner in SH-SY5Y cultured cells as compared to control cells (Figure 2A). This enhanced steroid production was interpreted as a result of self-defense probably to overcome harmful effects of Aβ.

The levels of free neurosteroids are also regulated in AD brain regions. Decreased PREG and DHEA concentrations were noticed in several brain areas of aged AD patients albeit not significantly different than controls. Similar steroid levels were found between regions in the AD group, including frontal cortex, striatum, amygdala and hippocampus [39]. In the studies by Marx et al. 2006 and Naylor et al. 2010 [41,42], high DHEA and PREG concentrations were observed in the prefrontal and temporal cortices of AD patients and tended to be positively correlated with Braak and Braak stage [41]. Similarly, in SH-SY5Y neuronal cells, treatment with Aβ peptides (Aβ_25–35_, Aβ_1–40_ or Aβ_1–42_) at toxic doses significantly enhanced PREG levels [40] (Figure 2B). PREG levels were higher in the group treated with Aβ_25–35_ than the two others and proportionate to cell cholesterol content. The author suggested that the effect of Aβ on PREG levels might be a result of self-defense. PREG concentration also significantly increased in the hippocampus on days 7 and 12 following bilateral injection of Aβ_25–35_ in the rat CA1 region [43].

Lower PROG concentrations were quantified in several brain regions (frontal cortex, striatum, hypothalamus and hippocampus) of AD patients than controls, with no significant difference between the groups possibly due to the low number of patients [39]. In rats, PROG levels were significantly reduced in the hippocampus and prefrontal cortex following prolonged bilateral administration of Aβ_25–35_ into the CA1 region [43]. Similarly, the synthesis of PROG from PREG was reduced in neuronal cell cultures under Aβ burden conditions [44,45] (Figure 2B).

Allopregnanolone was found to be significantly decreased in the prefrontal and temporal cortices of AD patients [41,42]. In contrast to PROG, allopregnanolone content was unchanged in the hippocampus of Aβ-treated rats nor was its production in SH-SY5Y cells displaying Aβ pathology [43,44,45]. These data suggest that Aβ may target only specific steroidogenic enzymes, decreasing 3β-HSD activity to reduce PROG without affecting 3α-HSD involved in allopregnanolone. Thus, steroid changes in AD brain and those under Aβ burden conditions appear inconsistent. The direct effects of Aβ peptides on steroid enzyme expression and activity need further evaluation in several in vivo models of Aβ pathology. The mechanism by which allopregnanolone decreased in AD brain remains unclear. He et al. 2005 [46] demonstrated that the human brain 17β-HSD type 10 can catalyze allopregnanolone oxidation to yield 5α-DHP. High levels of 17β-HSD10 were quantified in activated astrocytes of several brain regions with AD pathology, including the hippocampus, hypothalamus and amygdala [46]. The upregulation of 17β-SHD activity may then lead to local reduced allopregnanolone levels in AD brain. In fact, discrepancies were observed in 17β-HSD type 10 levels that were either upregulated in the late stages of the AD brain [26] or unchanged [47]. This mitochondrial enzyme may also interact with soluble or aggregated Aβ [48,49]. In addition, it is unclear whether allopregnanolone reduction in the AD brain is related to Aβ accumulation per se. The decrease in allopreganolone levels in the AD brain was found inversely correlated to Braak and Braak stage, reflecting neuropathological disease severity [41,42]. Thus, the allopregnanolone content in the AD brain may rather have relevance in tau pathology since Braak and Braak staging focus on NFTs. Interestingly, Luchetti et al. 2011 [47] detected high levels of 3α-HSD type 3 mRNA in cortical astrocytes starting from Braak stage 3. Although an enhancement in allopregnanolone synthesis was not demonstrated, it may be seen as a rescue mechanism early in the disease process aimed at promoting brain protection.

The relationship between pathological tau and neuroactive steroids remains elusive. Only one investigation stated that tau with pathogenic mutation P301L associated with frontotemporal dementia (FTD) had no impact on neurosteroid synthesis: neither the production of PROG nor allopregnanolone was modified in SH-SY5Y cells stably transfected by the mutant P301L tau and incubated with PREG as compared to native cells [44]. Further studies are required to deeply explore the relationship between pathological forms of tau (P301S mutant, oligomers or fibrillary tau) and neurosteroid concentrations.

### 2.3. Changes in Sex Steroids and Biosynthetic Enzyme Levels

**Figure 3:**
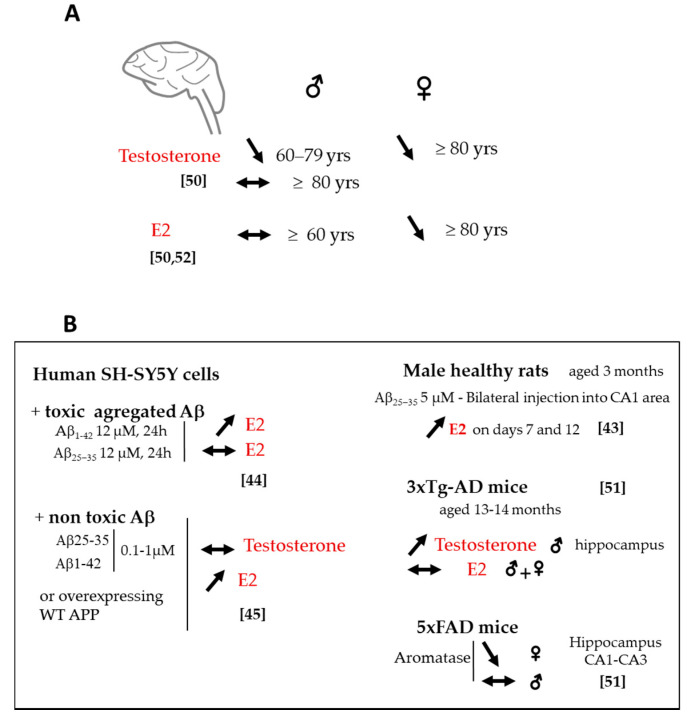
Change in endogenous sex-steroid levels. (**A**) In the AD brain compared to nondemented controls. (**B**) Under in vitro and in vivo Aβ burden conditions. ↓: decrease, ↑: increase, ↔: no change vs. Aβ.

Changes in sex steroid levels are also relevant to AD. The age-related decrease in brain levels of testosterone in men and E2 in women during menopause have been associated with greater risk of developing AD [37,50]. Brain testosterone levels were found to be lower in men with AD than control subjects. Rosario et al. 2011 [50] mentioned that testosterone was regulated according to age and disease stage (Figure 3A). It was reduced only in the brains of men aged 60–79 years diagnosed with AD at mild stages but not in those over 80, suggesting that testosterone loss in the brain occurs early in the disease process. Indeed, brain testosterone was inversely correlated with soluble Aβ levels [50]. Interestingly, Type 1 17β-HSD mRNA progressively increased in the AD prefrontal cortex, starting from Braak stages 3–4 suggesting an early increase in testosterone synthesis in relation with tau pathology that may culminate in Braak stages 5–6 [47]. In male 3xTg-AD mice, an increase in brain testosterone with age was seen in the hippocampus (Figure 3B) associated with the expression of the early tau pathologic conformational marker Alz50 and extra-neuronal Aβ deposition, with no change in the androgen receptor level [51].

In AD women, the regulation of brain E2 is also age-dependent (Figure 3A). Women with AD aged 80 years and older exhibited significantly lower brain E2 than age-matched nondemented controls [50,52] (Figure 3A). Surprisingly, aromatase expression increased from mild to moderate stages, particularly in the prefrontal cortex and hippocampus [47,53]. This aromatase increase is even higher in the later stages and occurred in both astrocytes [50] and neurons [53].

Again, these results point out the importance of the disease stage in the evaluation of steroid and enzyme levels in the AD brain. The enhancement of testosterone levels in the late stage of AD pathology as well as the upregulation of aromatase and 17β-HDS type 1 (which can also convert estrone to E2) to enhance E2 synthesis could be viewed as part of a compensatory neuroprotective mechanism. Consistent with this idea, brain injury in rodents rapidly upregulated aromatase enzyme expression in glial cells at the injury site suggesting that increased E2 levels may afford protection in injured neurons [54,55]. An upregulation of E2 synthesis was observed in SH-SY5Y neuronal cells with Aβ burden [44,45] (Figure 3B). E2 significantly increased in the prefrontal cortex and in the hippocampus after bilateral infusion of Aβ into the male rat hippocampus [43] (Figure 3B). Surprisingly, in aged 3xTg-AD mice, the increase in brain testosterone was not associated with a concomitant increase in brain E2. Contrariwise, brain E2 did not change with age in both males and females [51].

### 2.4. Sex Difference in Neuroactive Steroid Levels

Sex differences have been noted in AD but remain debated. Most studies suggest that women have greater frequency and lifetime risk than men. There is also mixed opinion concerning prevalence and incidence rates, and disease course [56,57,58,59]. Sex difference in the occurrence and distribution of Aβ plaques in the brain or CSF Aβ concentrations is unknown. Evidence of any impact of sex on brain tau hyperphosphorylation and NFTs or CSF tau levels is not established [60]. Sex differences in neuroactive steroid levels may be important to consider in AD, yet are poorly explored. The study by Corbo et al. 2014 [61] showed a direct influence of CYP17 genotypes on AD susceptibility and age of onset mainly in men. The study of Rosario et al. 2011 [50] revealed sex-specific brain levels of testosterone and E2 in AD patients (Figure 3A). In postmortem AD brain samples, reduced testosterone and E2 levels were noted in women older than 80 years, while only testosterone decreased in men aged 60–79 years. An earlier report from the same group indicated that E2 brain levels did not decrease with age in men and were unaffected in AD brain at any stage of the pathology [62]. Furthermore, the aromatase gene CYP19A1 polymorphisms appeared to be exclusively associated with AD risk in women and not in men [63,64]. Therefore, brain E2 and aromatase levels do not seem to be linked to AD status in men. It is interesting to note that sex-specific differences in the aromatase were demonstrated in transgenic mice that early express Aβ pathology (Figure 3B) [53]. The expression of aromatase mRNA and protein in the hippocampus was similar in male 5xFAD mice (that express human APP and PSEN1 transgene with a total of five mutations) and controls, whereas it was significantly lower in 5xFAD females [53]. Therefore, the contribution of E2 to the sex-specific effect seen in AD may primarily be related to Aβ pathology among AD etiologies. The regulation of E2 production and related enzymes (aromatase, 17β-HSD) in the brain of AD women and men may deserve further investigation that needs to take into account age, sex, and disease state.

Direct access to the human brain remains challenging as compared to CSF. Changes in CSF neuroactive steroids might reflect changes in the brain and be a good alternative to better understand their role in AD. Changes in CSF neuroactive steroids were previously observed in humans with no brain disorders [65,66,67], but they are scarce in AD. A lower CSF E2 level was found in AD female patients compared to nondemented ones [68] and this corroborates the lower brain E2 in AD women as compared to controls [50,52].

## 3. Steroids and Genetics of Late-Onset AD

The only strong and well-established genetic risk factor for the development of late-onset AD is the inheritance of the APOE-ε4 allele (for review [69]). ApoE is a multifunctional protein that binds to the low-density lipoprotein receptor family and therefore plays a central role in maintaining cholesterol/lipid homeostasis in the brain [70]. Recent studies provide evidence of a connection between abnormal cholesterol metabolism by ApoE4 and AD pathology. Impaired efflux of cholesterol in APOE-ε4 neurons contributes to its intracellular accumulation and Aβ increase [71]. Since cholesterol is the precursor of all steroids, associations with APOE4 and cholesterol-derived steroids could be considered. However, only a few reports have investigated this issue. A significant decrease in allopregnanolone levels was observed in the temporal cortex of patients positive for the APOE-ε4 allele compared to patients not carrying it [42]. Sex differences in the risk of AD are also modified by APOE genotypes. The APOE-ε4 risk of developing AD was thought to be greater in women as compared to male carriers [72,73]. In fact, greater risk of late-onset AD was evident in APOE-ε4 homozygote females, while increased risk of early-onset AD was evident in APOE-ε4 homozygote males [8,72]. Increased APOE-related risk in women seems to be associated with tau pathology [72]. Why APOE-ε4 gene confers higher risk in women is unclear. Whether E2 levels in APOE-ε4 carrying women are directly or indirectly linked to AD risk and severity is also uncertain. In men, E2 was lower in APOE-ε4 carriers with AD than controls, and testosterone was lower only in AD men without APOE-ε4 [74]. The roles of endogenous steroid levels as factors relevant to APOE-ε4 carriers at risk of AD remain to be fully characterized.

## 4. Protective Effects of Neuroactive Steroids on AD-Like Neuropathology

Endogenous neuroactive (neuro) steroids are among the most potent modulators of CNS functions. The changes in their levels in the AD brain suggest that they may be key modulators of AD-like neuropathology. They can target several important landmarks of AD pathology, via a variety of mechanisms including prevention of apoptosis, oxidative stress, mitochondrial dysfunction, synaptic loss and regulation of intracellular survival signaling pathways.

### 4.1. Amyloid-β Pathology

The Aβ protein is a crucial initiator that triggers the pathological events leading to AD through accumulation and aggregation within the CNS. It is a small protein composed of 39–43 amino acids generated by sequential cleavage of human amyloid precursor protein by β- and γ- secretases. Its two major forms are Aβ_1–40_ and Aβ_1–42_ with the latter being more prone to aggregate in AD patients. The Aβ_25–35_ fragment is a biologically active C-terminal region of Aβ_1–42_. Aggregated Aβ peptides have harmful properties, but proof implicates soluble oligomeric Aβ as the primary noxious form. Aβ toxicity to neurons promotes a myriad of detrimental cellular events associated with neuronal death including, for instance, pore formation, oxidative stress, lipid peroxidation, mitochondrial dysfunction, neuroinflammation, loss of synapses and disruption of the cytoskeleton, among others (for reviews [2,75]). All Aβ forms are convenient tools for in vitro and in vivo experimental models of Aβ pathology associated with AD. Several steroids illustrated their capacity to protect cells from death induced by these peptides and, the way they achieve their neuroprotective effects is elucidated most of the time. We may note that, for a given steroid, results may appear controversial depending on the Aβ form, cell type, dose and duration of steroid treatment used. Inversely, Aβ peptides can affect steroid levels in neuronal cells.

#### 4.1.1. Effects of Neurosteroids on Aβ Toxicity

**Figure 4:**
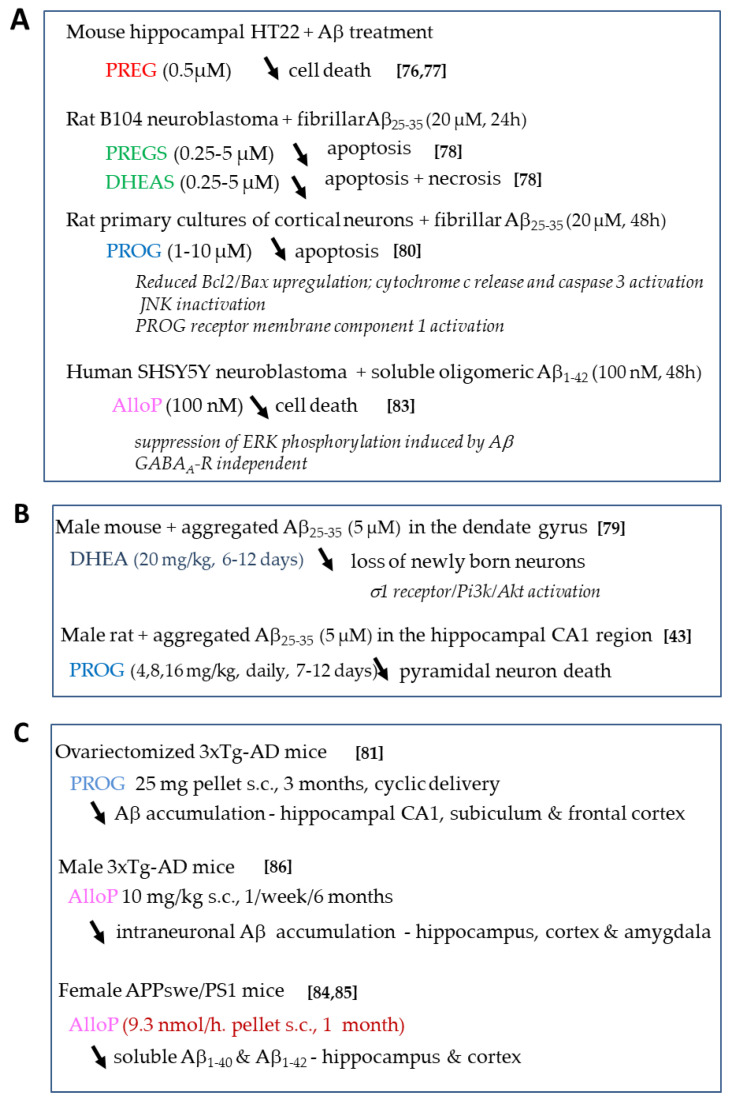
Protective neurosteroid treatment on Aβ toxicity. (**A**) in vitro (**B**) in vivo after Aβ administration in rodent hippocampus and (**C**) in vivo in transgenic mouse models of AD.

The effects of neurosteroids on Aβ toxicity are summarized in Figure 4. Regarding PREG and PREGS, very few studies have reported their regulatory effects on Aβ-induced neuronal toxicity. PREG protected mouse hippocampal (HT-22) and rat pheochromocytoma (PC-12) cell lines in a dose-dependent and significant manner against Aβ_25–35_-induced cell death [76,77], but the molecular target(s) for its action awaits identification.

Interestingly, PREGS treatment differentially regulated neuronal cell survival in in vitro Aβ-induced AD models. One study indicated that it did not have any effect on the Aβ_25–35_-induced decrease in PC12 cell viability [77]. More recently, we observed that it significantly and dose-dependently counteracted the reduced cell viability induced by Aβ_25–35_ in rat B104 neuroblastoma cells by preventing the cells from entering late apoptosis [78]. DHEAS was also capable of significantly attenuating Aβ_25–35_-induced toxicity, by preventing the cells from entering both late apoptosis and necrosis [78] (Figure 4A)]. DHEA neuroprotection was observed against neurite growth impairment and loss of newborn neurons caused by Aβ_25–35_ infusion in the mouse dentate gyrus. This effect involved a sigma1 receptor-dependent activation of PI3K-Akt-mTOR signaling pathways that play a role in the regulation of apoptosis and cell growth [79] (Figure 4B).

PROG has also been shown as an effective neuroprotectant in AD models (Figure 4). Several studies stated that PROG exerts neuroprotective effects against Aβ_25–35_-induced toxicity in vitro and in vivo (Figure 4A,B). PROG significantly and dose-dependently improved neuronal survival in primary cultured rat cortical neurons treated by Aβ_25–35_ [80]. This effect implicated a decrease in the upregulation of the apoptotic marker Bax/Bcl2 ratio that signals the loss of mitochondrial membrane potential and downstream caspase-3 activation. In addition, the mitochondrial PROG receptor membrane component 1 was activated and the c-Jun N-terminal kinase pathway inactivated (Figure 4A). The classic PROG receptor was also partly involved. In vivo, PROG treatment reduced the decrease in hippocampal cell number induced by Aβ_25–35_ injection into the rat hippocampal CA1 region [43] (Figure 4B). The delivery procedure had a differential impact on the effect of PROG on Aβ intraneuronal accumulation. Indeed, in ovariectomized triple transgenic AD mice 3xTg-AD (bearing the human APP_SWE_, Tau_P301L_, and PS1_M146V_ genes linked to AD and FTD), PROG cyclic delivery significantly attenuated Aβ accumulation in different brain regions (Figure 4C), whereas PROG continuous treatment for three months was devoid of any action [81]. These findings highlighted the importance of timing and duration of steroid administration. Additional studies are needed to clarify the mechanisms underlying different PROG treatment outcomes. One possible mechanism of PROG positive result may involve enhancement of Aβ clearance factors [82].

The neuroprotective effects of allopregnanolone against Aβ pathology have also been demonstrated. Allopregnanolone prevented the neurotoxicity resulting from Aβ_1–42_ exposure in SH-SY5Y and primary cortical neurons via the suppression of extracellular signal-regulated kinase phosphorylation induced by Aβ and independently of GABA_A_ receptor activity [83] (Figure 4A). Discrepancies were noted in the in vivo effects of allopregnanolone treatment on Aβ levels across AD transgenic mouse models that depended on the duration, frequency and time window of treatment. For instance, short chronic allopregnanolone treatment in two models of autosomal dominant AD resulted in increased soluble Aβ levels in the brain of female APPswe/PS1 mice but not in female APPswe/Arc (Figure 4C) [84,85]. This difference might be explained by the distinct contribution of mutated PS1 and Arc genes in regulating Aβ production. Whether these data have any relevance to human late-onset AD is open. In male 3xTg-AD mice, an allopregnanolone regimen of one/week/six months at an early stage of pathology progression showed the highest efficacy on Aβ reduction as compared to three/week/three months and one/month [86]. Therefore, the therapeutic time window is important for steroid efficacy and intra- not extraneuronal Aβ seems to be a critical target for allopregnanolone benefits.

#### 4.1.2. Effects of Sex Steroids on Aβ Toxicity

**Figure 5:**
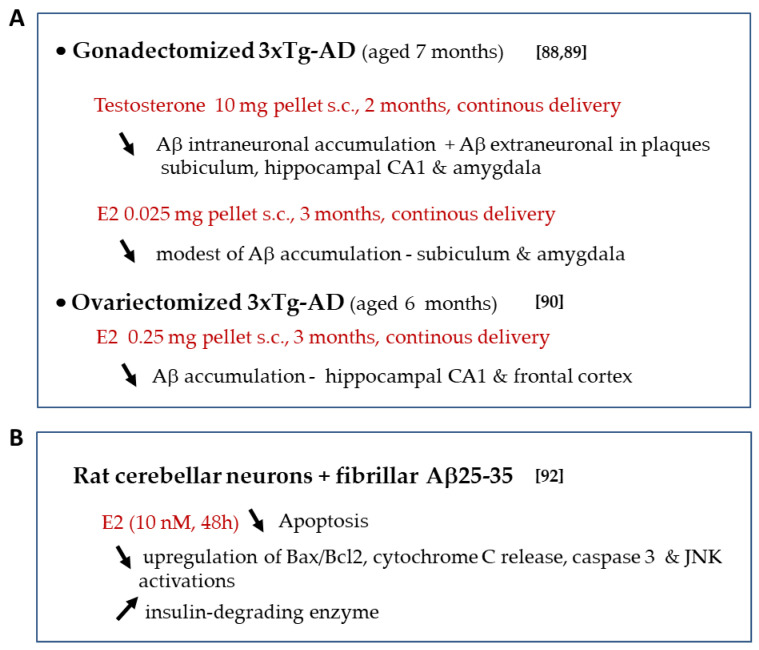
Sex-steroid treatment on Aβ toxicity in vivo (**A**) and in vitro (**B**). ↓: decrease, ↑: increase, ↔ no change.

Androgens may contribute to slowing down AD. Gonadectomy increased Aβ levels and androgens exerted a substantial inhibition of Aβ accumulation in male AD mouse models [87,88]. Testosterone treatment of gonadectomized 3xTg-AD mice prevented the increase of Aβ accumulation in several brain regions by direct activation of the androgen receptor [88,89]. E2 treatment was partially effective in reducing Aβ in these mice. Both testosterone and E2 reduced tau hyperphosphorylation [89], suggesting a role of testosterone via aromatization (Figure 5A).

The role of E2 in reducing Aβ accumulation and associated toxic events has been investigated [90]. In ovariectomized 3xTg-AD, E2 treatment entirely prevented Aβ accumulation in specific brain regions [91] (Figure 5A). E2 protective effect was partially attenuated by PROG suggesting that these steroids act in part via a common mechanism that yet needs clarification. E2 also prevented the Aβ-induced apoptosis in rat cerebellar granule cells [92] (Figure 5B). The protective effects of E2 against Aβ involved the prevention of Bax/Bcl-2 ratio up-regulation, subsequent cytochrome c mitochondrial release and caspase 3 activation [92]. They could also be associated with an increased expression of the insulin-degrading enzyme involved in Aβ clearance [82] as well as enhanced Aβ proteases and somatostatinergic systems [93]. Intriguingly, E2 actions occurred independently of classical estrogen receptor mechanisms. Of note, previous studies indicate that brain E2 deficiency accelerated Aβ deposition in APP23 mice (overexpressing the human APP751 with familial Swedish AD double mutation KM670/671NL) [52]. This report highlighted the potential protective role of local E2 levels in the female brain as compared to chronic ovarian hormone deprivation.

### 4.2. Tau Pathology

Research on tau protein revealed that it undergoes several changes in the AD brain, mainly hyperphosphorylation, truncation, aggregation, seeding and spreading. Tau oligomers, fibrils and aggregates constitutive of NFTs exert neurotoxicity in AD. Hyperphosphorylated tau (PHF-tau) is well-described and closely connected with neurodegeneration and cognitive dysfunction in AD (for review [94]). In contrast to Aβ, the relationship between tau and steroids is still in its infancy.

We previously demonstrated that hyperphosphorylated tau levels (recognized by AT8 antibody) were negatively correlated with DHEAS concentrations in the hypothalamus of aged AD patients as compared with nondemented ones [39]. In patients with FTD, low amounts of PROG in the serum significantly correlated with low disease severity [95], but the link with brain PROG was lacking. Interestingly, some studies reported the effects of neuroactive steroids on physiological and pathological tau levels. For instance, PROG treatment significantly decreased total tau in human neuroblastoma SK-N-MC cell line [95]. In ovariectomized 3xTg-AD mice, both cyclic and continuous PROG treatment (25.0 pellet s.c. for three months) significantly attenuated hyperphosphorylated tau levels (recognized by AT8 antibody) in the hippocampus and cortex, as compared to control mice [81,91]. The way in which PROG exerts its protective effect on tau hyperphosphorylation is unknown.

Testosterone treatment (10 mg pellet s.c. continuous delivery for two months) reduced tau hyperphosphorylation (AT8) in the CA1 hippocampus gonadectomized male 3xTg-AD mice to levels lower than observed in controls, independently of androgen receptor activation [89]. The influence of E2 on AD-like tau pathology has been described, but discrepancies have been noted depending on the in vitro and in vivo models. E2 treatment sustained tau hyperphosphorylation induced by protein kinase A activation in human embryonic kidney HEK293 cells stably expressing tau441 [96]. Similarly, it enhanced tau phosphorylation on several epitopes (Ser396, Ser262 but not Ser202/Thr205 (AT8)) through adenosine monophosphate protein kinase activation human SH-SH5Y neuroblastoma cells [97]. In contrast, it attenuated tau hyperphosphorylation at multiple sites (S396/404, Thr231, Thr205, S199/202) induced by transient GSK3β overexpression in mouse N2A neuroblastoma cells [98]. In ovariectomized 3xTg-AD, E2 individual treatment was ineffective on tau phosphorylation whereas combined E2 and PROG treatment reduced phosphotau (AT8) levels [81]. Whether sex steroids regulate other pathological forms of tau such as tau oligomers deserves further investigation. Recently, the impact of sex on tau pathology was mentioned in the P301L model of FTD, with female mice displaying significantly higher total tau and phosphotau levels (AT8 and AT100 (ptau-T212/S214) positive neurons) in the cerebral cortex and hippocampus than males [99]. It is likely that the severity of sex-related tauopathy depends partly on local sex steroid levels, but this remains to be shown. One earlier exploratory study in women with FTD aged 70 year-old indicated a high correlation between estrogen use and the development of FTD, suggesting that estrogen replacement therapy may be contraindicated in women with early FTD symptoms [100].

### 4.3. Mitochondrial Impairment

Mitochondria are highly dynamic organelles essential for the bioenergetic state of the cell. Mitochondrial dysfunction results in decreased energy production, weakened respiratory function, altered calcium homeostasis, oxidative stress or neuroinflammation that leads to neuronal death. Mitochondrial defects are features of both sporadic and familial AD. It is known that Aβ and hyperphosphorylated tau negatively impact mitochondrial function. Alternatively, mitochondrial dysfunction can generate Aβ accumulation or be independent of Aβ (for review [101]).

Consequences of mitochondrial dysfunction, particularly oxidative stress, can influence brain steroid levels and steroidogenic pathways and therefore may have an implication in AD. Treatment with ferrous sulfate-catalyzed oxidative neuronal damage caused a significant increase in DHEA levels in the hippocampus and frontal cortex of AD patients [102]. The authors indicated that DHEA production may come from an oxygenated metabolite of PREG or cholesterol. It was unclear if this endogenous mechanism was able to rescue neurons from death. Low DHEAS in aged rat brain was found to be associated with reduced antioxidant glutathione [103]. This result may have implication in AD pathology since reduction in brain glutathione content is a prominent feature of the disease [104]. Upregulation of glutathione or DHEAS levels is a potential strategy to consider for slowing down AD progression [105]. In addition, sex steroid loss and sex differences are linked to the mitochondrial dysfunction. Impaired mitochondrial function was found in female 3xTg-AD brains at a late stage (nine months and onwards) of AD-like disease while at an early stage (around one month) in males [106]. Furthermore, chronic ovarian hormone deprivation by ovariectomy in 3xTg-AD mice exacerbated brain bioenergetics deficits [107].

Multiple approaches have been undertaken to target mitochondrial abnormalities for developing neuroprotective strategies. Regarding neurosteroids, they have been shown to display varied antioxidant effects against oxidative stress-induced cell death caused by Aβ (Table 1).

DHEA treatment of isolated mouse brain mitochondria attenuated the decreased mitochondrial respiration and increase in reactive oxygen species (ROS) production induced by Aβ peptide [108]. Allopregnanolone significantly diminished the intracellular production of ROS, enhanced superoxide dismutase (SOD) activity involved in Aβ-induced PC12 cell death [109]. Notably, we previously demonstrated the antioxidant neuroprotective effects of the synthetic enantiomers of PREGS and DHEAS, which prevented aβ_25–35_-induced lipid peroxidation in the mouse hippocampus [78]. Whether PROG regulates AD-induced mitochondrial dysfunction is still unknown, but its benefits in normal brain mitochondria include enhancing functional efficiency and increased metabolic rates [111]. The effects of PREG, DHEA and their sulfated derivatives on mitochondrial deficits are currently lacking.

A complex relationship exists between sex steroids and mitochondrial function that depends on sex, aging or disease (for review [112]). In AD, the role of E2 on the maintenance and function of mitochondria is ambiguous [113]. A selection of studies demonstrated the neuroprotective effects of E2 against AD-related mitochondrial injury. E2 pretreatment (10 nM, 24 h) protected against the heavy metal (cobalt and mercury) induced oxidative stress (loss of glutathione) and the increase in Aβ_1–40_ secretion in SH-SY5Y neuroblastoma cells [114]. E2 pretreatment (100 nM and 1 µM, 2 h) preserved mitochondrial membrane potential and intracellular Ca2+ homeostasis, attenuated ATP depletion, and reduced mitochondrial calcium overload induced by H_2_O_2_ (150 µM) toxicity in human neuroblastoma SH-N-SY cells [115]. E2 pretreatment protected against Aβ_1–42_-induced generation of ROS in choroid plexus explants and cultured choroid plexus epithelial cells through the E2 receptor-dependent internalization of reduction of Aβ uptake [110] (Table 1). Notably, E2 exacerbation of oxidative stress-induced-cell death was recently unveiled in C6 glial cells and N27 neuronal cells and explained by the time window, i.e., deleterious steroid post-treatment vs. beneficial steroid pretreatment [116]. E2 treatment of ovariectomized 3xTg-AD mice prevented the decrease in mitochondrial respiration, increase in oxidative stress and the subsequent Aβ-accumulation in brain induced by ovariectomy [107] (Table 1). Of interest are the few studies attesting for a sex-steroid difference in oxidative stress markers in AD patients. For instance, the higher activities of superoxide dismutase and glutathione peroxidase in postmortem AD brain compared to controls were further upregulated in women than men [117,118].

### 4.4. Neuroinflammation

Neuroinflammation was historically considered as a secondary event in neurodegenerative diseases. Accumulating evidence now suggests that it is a key mechanism in AD initiation and progression. Abnormalities of Aβ and tau cause activation of microglia and astrocytes, and trigger the innate immune system by releasing proinflammatory mediators (for reviews [119,120]). Recent studies have revealed different types of pathological microglia associated with AD and key regulators of the microglia pathogenicity, such as the triggering receptor expressed on myeloid cells 2 [121,122]. Therefore, glial activation with associated inflammatory mediators and regulators are important targets for therapeutic approaches in AD research.

Few studies have addressed the role of neurosteroids in preventing neuroinflammation in AD with an impact on Aβ-induced increase in cytokine secretion and microglial activation. PROG blocked Aβ_25–35_-mediated upregulation of TNFα and interleukin-1 (IL-1) and concomitantly increased cell survival in rat hippocampus [43]. PROG suppressed inflammatory responses induced by oligomeric Aβ_1–42_ in primary astrocyte cultures, including IL-1 β and TNF-α production. The reduction of endoplasmic reticulum stress markers (PERK/elF2α) triggered by Aβ was believed to be part of the PROG anti-inflammatory action [123].

Strong interactions between sex steroids and chronic inflammation were evidenced in AD (for review [124]). Physiological concentrations of E2 and testosterone displayed anti-inflammatory activities in several AD models (Table 2). In APP23 transgenic mice (overexpressing human APP751 with the familial Swedish AD double mutation), ovariectomy led to more Aβ plaques associated with reactive microglia that parallel disease progression. Chronic E2 administration delayed this process by directly acting on resident microglia helping them to remove to Aβ [125]. In a primary culture of microglia derived from human cortex, E2 treatment enhanced uptake of Aβ [126]. The E2 anti-inflammatory effect was likely to be mediated via the decrease of the Aβ-induced NF-κB activation, which is critical for the induction of inflammatory response genes in activated BV-2 microglial cells [125,127]. Testosterone and its 5α-reduced metabolite 5α-dihydrotestosterone promote microglia to phagocytose and clear Aβ and inhibit proinflammatory cytokine expression in Aβ-activated murine microglia cell cultures (Table 2). Androgen administration also reduced Aβ_1–42_-induced IL-1β expression and neuronal death in the murine hippocampus. These anti-inflammatory effects were mediated by the suppression of aβ-induced NF-κB and p38 mitogen-activated protein kinase activation [128]. In addition, 5α-dihydrotestosterone promoted Aβ uptake by microglia through increasing formyl peptide receptor 2 (FPR2) and enhanced Aβ clearance by increasing the levels of the Aβ degrading enzyme endothelin-converting enzyme 1c (Table 2) [128]. Thus, the anti-inflammatory activities of E2 and testosterone contribute to their protective effects on Aβ-activated microglia-induced neurotoxicity, independently of their respective classical receptors. Direct regulation of resident microglia by steroids inhibiting chronic inflammation associated with AD may be a valuable therapeutic strategy of AD.

Only limited studies have focused on tau pathology and neuroinflammation (for review [129]). Tau-mediated neuroinflammation and disease was evidenced in AD patients and models of pure tauopathy. For instance, positron emission tomography (PET) analysis of AD patient brains showed a direct positive correlation between tau aggregation and microglial activation in the parahippocampus at the early stage of the disease [130]. Activated microglia/infiltrating macrophages as well as induction and overproduction of inflammatory mediators (IL-1β and cyclooxygenase-2) were detected in the hippocampus and cortex of P301S transgenic mice and in the brain a patient with FTD associated with P301S mutation [131,132]. In fact, distinct microglial responses were observed depending on the type of tau pathology, tau phosphorylation state and it is even believed that reducing microglia number does not change tau pathological lesions [133,134,135]. In this context, the role of neuroactive steroids in tau-mediated neuroinflammation awaits further experimentation.

### 4.5. Neurogenesis, Synaptic Failure and Memory Loss

Neuronal loss in AD leads to progressive brain atrophy. Neurogenesis, which allows the endogenous formation of newly born neurons in the adult brain, is known to be less efficient during aging and in neurodegenerative diseases. Reduced neurogenesis in AD is associated with the lack of maturation and functional integration of newborn neurons in late stages of the disease [136]. Therefore, stimulating endogenous neurogenesis could be a therapeutic target for early intervention in AD. The neurosteroid modulation may have possible benefits on the course of the disease by increasing survival of adult-born neurons contributing to memory improvement.

PREGS significantly reduced the impairment of neurite growth as well as survival and maturation of hippocampal dentate gyrus newborn neurons of APPswe/PS1dE9 mice [137]. Several studies in the laboratory of Brinton D. demonstrated the efficacy of allopregnanolone in promoting neurogenesis in the hippocampal subgranular zone of 3xTg-AD mice [86]. The regenerative action of allopregnanolone involved GABA_A_-R activation inducing chloride ion efflux from neural progenitor and upregulation of cell cycle genes that promote mitosis and repress cell division [138]. PREGS and DHEAS neurotrophic effects were observed in cultured rat neuroblastoma cells by the significant reduction of Aβ_25–35_-induced decrease of neurite growth [78].

Sex steroids are also important players in regulating adult hippocampal neurogenesis. There is ample evidence that E2 and testosterone enhanced the hippocampal dentate gyrus neuronal renewal in young adult female and male rodents, resulting in increased memory function (for reviews [139,140]). By contrast, literature highlighting their impact on neurogenesis in AD models is poor. One study indicated that chronic E2 administration significantly increased hippocampal neurogenesis in ovariectomized mice injected with Aβ_1–42_ into the brain. This E2 protective effect occurring during the early, not late, stage of the Aβ pathological process, alleviated memory loss [141]. The effects of androgens on neurogenesis in AD models are yet to be discovered.

Synaptic impairment in the neocortex and hippocampus is an early pathological feature of AD that correlates strongly with cognitive decline. Multiple studies support that pathological Aβ and tau affect the integrity of synaptic function individually and in interaction with several complex mechanisms that lead to impaired synaptic plasticity, neurotransmitter receptor dysfunction and memory loss (for reviews [3,142]). Thus, preventing synaptic dysfunction may be an attractive approach to delay AD progression and symptoms.

Under physiological conditions, neuroactive steroids play a significant role in the integrity of synapses and the modulation of synaptic plasticity underlying learning and memory. PREGS is a well-known modulator of glutamatergic excitatory synaptic transmission and plasticity. It sustains presynaptic glutamate release, potentiates glutamatergic post-synaptic N-methyl-D-aspartate receptor (NMDA-R) function, induces its trafficking and enhances long-term potentiation [143,144]. Allopregnanolone is a potent allosteric modulator of GABA_A_ receptor activity thereby exerts control over neuronal excitability. Recently, it was shown to increase mature excitatory synapse dendritic spines of cultured mature hippocampal neurons [145]. E2 regulated excitatory synaptic transmission in hippocampal neurons via estrogen receptor activation and by altering synaptic distribution of NMDA-Rs in prefrontal cortex [146,147]. Testosterone also enhanced genesis of spines through androgen receptor activation [148,149].

Surprisingly, the modulation of synaptic function and plasticity by steroids in AD is almost unexplored. One report highlighted the beneficial effects of E2 on early-Aβ_25–35_-induced synaptic dysfunction in organotypic hippocampal cultures [150]. Testosterone improved the oligomeric Aβ-induced presynaptic failure in primary cultures of hippocampal neurons [151]. Sex impact on postsynaptic protein levels was shown in P301L mice, and a more severe synaptopathy observed in females than males [99]. The potential protective effects of neuroactive (neuro)steroids on synaptic dysfunction associated with Aβ and tau pathology needs to be widely explored.

Tremendous efforts have been devoted to characterizing the progressive impairment in learning and memory in the course of AD. Early stages of AD are marked by salient deficits in episodic and working memory as well as novelty processing impairment [4,152]. Although animal AD models do not fully recapitulate human AD cognitive deficits, they are a very useful tool for assessing memory functioning and pharmacological intervention in behavioral tasks [153].

Evidence demonstrates that neuroactive steroids differentially regulate learning and memory performance in rodents. The memory deficits induced by aβ_25–35_ acute central administration in young adult male mice were attenuated by PREGS and DHEAS in the spontaneous alternation, passive avoidance or Morris water maze tests [78,154] (Table 3). The PREGS and DHEAS protective effects against Aβ_25–35_-induced amnesia involved the activation of sigma1 and α7 nicotinic acetylcholine receptors [154,155]. Interestingly, the synthetic enantiomeric analogues of PREGS and DHEAS also behaved as antiamnestic molecules against Aβ_25–35_ in young adult mice [78]. Acute PROG treatment was devoid of activity per se in this model but prevented the antiamnesic effect of natural PREGS and DHEA through sigma1 receptor modulation [154]. By contrast, chronic PROG administration restored the spatial/hippocampal memory deficits in mid-age mutant female APPswePSEN1 Δe 9 mice tested in the object placement and water maze tasks. This improvement involved increased PROG metabolism towards allopregnanolone in the cerebral cortex but not in the hippocampus of transgenic mice [156]. Curiously, chronic allopregnanolone in the same APPSwe/PS1 mouse model caused memory impairment in males, not females, with an accelerated disease progression [84,85]. In mid-aged 3xTg-AD mice, not aged, allopregnanolone single injection remarkably restored hippocampal associative learning that depended on the survival of newly generated neurons in the dentate gyrus [157]. The underlying mechanisms of allopregnanolone age and sex-dependent action on AD-related memory impairment need further research.

The influence of sex steroids on memory function in humans and rodents has been comprehensively reviewed in literature. However, a great diversity of outcomes has been obtained (i.e., improvement, decrease or no effect) depending on the disease stage, subject gender, study design, modes of delivery, type of memory evaluated and steroid dosage, among others. This is true for androgenic neuroactive steroids DHEA, DHEAS, testosterone and for estrogen (for reviews [158,159,160]).

## 5. Modulation of Endogenous Neuroactive Steroid Production for Protection in AD

Restoration of steroid homeostasis could be achieved by the supplementation of neuroactive steroids with a proper dosing and treatment regimen. Nowadays, an innovative strategy that is more and more attractive is to restore altered endogenous levels of protective steroids by promoting neurosteroidogenesis. In this context, targeting the 18 kDa translocator protein (TSPO) is becoming a valuable strategy [161]. TSPO is a high-affinity cholesterol-binding protein involved in the intramitochondrial cholesterol transport and steroid biosynthesis [162]. It is predominantly expressed in steroid-synthesizing organs including brain, and TSPO ligand treatment increases concentrations of several steroids. For instance, the pharmacodynamic study recently conducted by our group revealed that TSPO activation by etifoxine stimulated steroidogenesis in male rat brain, preferentially towards the synthesis of PREG, PROG and allopregnanolone [163], suggesting a differential regulation of neurosteroid production by this TSPO agonist.

Moreover, TSPO is upregulated in reactive glial cells during CNS pathologies, including AD [164,165]. It is involved in the control of many fundamental functions including mitochondrial respiration and permeability, energy metabolism, cell proliferation and differentiation. Several TSPO ligands have proven their efficacy as neuroprotective, anti-inflammatory and regenerating molecules in experimental AD models [166]. The TSPO ligand Ro5-4864 (4′-chlorodiazepam) was neuroprotective against Aβ_1–40_-induced neurotoxicity in SH-5YSY neuroblastoma cells. It reduced the Aβ-induced apoptotic Bax upregulation and downregulation of survivin, a member of the inhibitor of apoptosis protein family [167]. In human SH-SY5Y neuroblastoma cells transfected with wild-type APP, TSPO ligand treatment (10 nM, 24 h) including two TSPO ligands of reference XDB173 and SSR-180,575 and two new imidazoquinazolinone compounds exerted neuroprotective effects. All compounds were able to improve mitochondrial respiration, decrease ROS and Aβ level while enhancing PREG synthesis [168]. In SH-SY5Y cells expressing the pathological tau-P301L and therefore abnormal tau hyperphosphorylation, a treatment with the same TSPO ligands (10-100 nM, 24 h) increased mitochondrial bioenergetics (ATP levels and mitochondrial membrane potential) [169]. They also improved the production of PREG at 20 µM at 2 h [169]. Furthermore, TSPO ligand efficacy was proven in in vivo AD models. Ro5-4864 (3 mg/kg i.p. once weekly for three months) in young and aged male 3xTg-AD mice improved memory loss (performance in the spontaneous alternation test) while attenuating hippocampal Aβ accumulation and gliosis [170]. Steroid synthesis occurred in the brain following Ro5-4864 injection (3 mg/kg i.p. once weekly beginning two weeks after surgery and continuing for four weeks) in gonadectomized 3xTg-AD mice. In young adults, PROG and testosterone levels significantly decreased in the brain whereas those of PREG allopregnanolone were not significantly affected [170]. In aged 3xTg-AD mice, Ro5-4864 did not significantly alter brain levels of testosterone; however brain levels of PROG and allopregnanolone decreased and brain PREG were unchanged [170]. PK11195 treatment (3 mg/kg i.p. once weekly for five weeks) in aged female 3xTg-AD mice improved memory (performance in the spontaneous alternation test) while reducing both soluble and aggregated Aβ [171].

## 6. Concluding Remarks

Protective strategies that increase neural functioning as well as attenuating multiple aspects of AD-related neuropathology are more than welcome for treating AD. Preclinical evidence indicates that neurosteroids and sex steroids can promote neuronal survival, neurogenesis and memory function, by limiting apoptosis, oxidative stress, mitochondrial failure and microglial activation. In some cases, steroid beneficial effects depend on sex and stage of the neuropathology process. TSPO ligands also confer protection against AD-related pathology and they are worthy of constant research in the context of AD treatment. Studies still need improvement by answering questions with regard to the true model with AD fully represented, the therapeutic time window of intervention as well as solutions for reproducing molecule efficacy and safety. In addition, our knowledge of neurosteroids, steroid enzymes and metabolism in the AD brain and CSF during the course of the disease is far from complete, and larger studies using standardized validated protocols are required. Revitalizing research on steroids in AD with novel concepts close to human condition may help to obtain better and successfully translated benefits in patients with AD. So far, the translation of basic research on steroids in AD has not been fruitful. Gaps are yet to be filled regarding appropriate steroid formulation, dosing and regimen, alone or in combination, administration routes and bioavailability. Also, because of sex differences in AD pathology and outcomes, there is an urgent need for new sex-specific, even personalized, steroid-based therapies. As the AD pathogenesis starts decades before symptoms appear, the question remains as to the earliest possible ethical intervention.

## Figures and Tables

**Table 1:** Protective effects of neuroactive steroids on Aβ-induced mitochondrial impairment.

DHEA	Allopregnanolone	E2
In vitro Forebrain mitochondria from male Swiss mouse + Aβ_1–42_ (4 µM) or Aβ_25–35_ (50 µM) for 20 min DHEA (3, 10 or 30 µM, 20 min) ↓ mitochondrial respiration dysfunction through σ1 receptor mechanism ↓ increased ROS production [108]	In vitro PC12 cells + aggregated Aβ_25–35_ (20 µM, 24 h) Allopregnanolone pretreatment (10 µM, 2 h) ↓ ROS generation ↓ SOD activity [109]	In vitro Choroid plexus explants or cell line Z310 + Aβ_1–42_ (0.66 µM, 24 h) E2 pretreatment (1 µM, 8–12 h)↓ ROS production, ↓ Aβ uptake [110] In vivo Ovariectomized 3xTg-AD mice E2 treatment (0.25 mg continuous 90-day pellet) prevented in isolated forebrain mitochondria: ↓ respiration, ↓ energy deficits ↓ Aβ load, ↓ lipid peroxidation [107]

↓: decrease ↑: increase vs. Aβ condition.

**Table 2:** Neuroactive steroid protective effects on Aβ-induced neuroinflammation.

Steroid Treatment	Models of Aβ-induced Inflammation	Inflammatory Response against Aβ Neurotoxicity
PROG Pretreatment 4, 8 or 16 mg/kg, i.p. daily, for 7 and 12 days after Aβ injection	In vivo Male rats + aggregated Aβ_25–35_ 5 µM in the hippocampal CA1 region	↓ the upregulation of TNFα and IL-1β induced by Aβ [43] ↓ endoplasmic reticulum stress markers PERK/elF2α
E2 pretreatment pellets 0.01 mg s.c. daily, from 5 to 10–14 months of age E2 pretreatment 100 nM, 48 h E2 pretreatment 10 µM, 60 min	In vivo Ovariectomized APP23 mice, 10–14 months of age (early stage of disease) In vitro Human cortical microglia + fluorescein-Aβ_1–42_ 100 nM Microglial BV-2 cell + aggregated Aβ_1–42_ 1 µM	↓ Mac-1 positive inflammatory plaques ↑ microglia clearance of Aβ [125] ↑ microglia uptake of Aβ, through non-classical estrogen receptor [126] ↓ Aβ-induced NF-κB [125,127]
Pretreatment Testosterone 100 nM Dihydrotestosterone 10 nM Pretreatment Testosterone 200 µg Dihydrotestosterone 100 µg s.c. every day for 2 weeks Pretreatment Dihydrotestosterone0.5 and 1 nM for 6 h	Murine microglia N9 cell line + aggregated Aβ_1–42_ (2 µM) for 30 min Male C57BL/6 mice + aggregated Aβ_1–42_ (1 µM) into the CA1 region Murine microglia N9 cell line + aggregated Aβ_1–42_ 1 µM for 1 h or 24 h	↓ Aβ-induced proinflammatory cytokine IL-1β via suppression of NF-κB and p38 activation by Aβ [128] ↓ Aβ-induced proinflammatory cytokine IL-1β [128] ↑ microglia Aβ uptake through upregulating FPR2 ↑ microglia clearance of Aβ through upregulating ECE-1c [128]

↓ decrease, ↑ increase vs. Aβ condition.

**Table 3:** Effects of neuroactive steroids on impaired neurogenesis and memory in AD models.

Impaired Neurogenesis	Aβ-Induced Memory Loss
• Male APPswe/PS1dE9 mice PREGS ↑ survival & maturation of newborn cells [137] Allopregnanolone 10 mg/kg 1/week/6 months at 3 months of age ↑ survival of newly generated cells in hippocampal subgranular zone [86] • Rat B104 neuroblastoma cells PREGS or DHEAS 5 µM, 24 h ↓ Aβ_25–35_ (20 µM)-induced decrease in neurite outgrowth [78] • 3 xTg-AD mice aged 6 and 9 months Allopregnanolone 10 mg/kg s.c. ↑ neurogenesis (BrDU^+^ neural progenitor survival) 1 h later [157]	• Male healthy mice + aggregated Aβ_25–35_ (3 nmol i.c.v) PREGS, DHEAS, DHEA (5, 10, 20 mg/kg s.c. respectively) ↓memory Aβ-induced deficits in short-term memory (spontaneous alternation in the Y-maze test) and in long-term memory (step-down passive avoidance test), 7 and 14 days post-Aβ infusion, respectively, through σ1 receptor activation [154] • Male healthy mice + aggregated Aβ_25–35_ (9 nmol i.c.v) Pretreatment by PREGS or DHEAS 0.5 nmol i.c.v 6 h before Aβ infusion ↓ memory deficits in short-term memory (spontaneous alternation test) and in long-term memory (step-through passive avoidance test) [78] • Ovariectomized APPswe/PS1dE9 mice PROG pellet (25 mg, 90-day release) ↑ short-term memory performance (object recognition T-maze test) [156] Allopreganolone (4.7 nmol or 9.3 nmol) for 12 weeks ↑ learning deficits in the Morris water maze [84] •3 xTg-AD mice aged 6 & 9 months Allopregnanolone 10 mg/kg s.c. 7 days prior to the start of the learning trials restored maximal learning capacity in the trace eyeblink conditioning, except at 12 months of age [157]

↓ decrease, ↑ increase vs. Aβ condition.

## Abbreviations

AD	Alzheimer’s disease
ApoE	apolipoprotein
APP	amyloid precursor protein
Aβ	Amyloid-β
CNS	central nervous system
CSF	cerebrospinal fluid
DHEA	dehydroepiandrosterone
DHEAS	dehydroepiandrosterone sulfate
E2	17β-estradiol
FTD	frontotemporal dementia
GABAA-R	γ-aminobutyric acid type A receptor
GC-MS	gas chromatography-mass spectrometry
IL-1β	interleukin-1β
mTOR	mechanistic target of rapamycin
NF-κB	nuclear factor-kappa B
NTFs	neurofibrillary tangles
P450scc	P450side chain cleavage
PI3K	phosphoinositide 3-kinase
PREG	pregnenolone
PREGS	pregnenolone sulfate
PROG	progesterone
PS1	presenilin-1
ROS	reactive oxygen species
TNFα	tumor necrosis factor alpha
TSPO	18 kDa translocator protein
yrs.	years
3 xTg-AD	triple transgenic mouse bearing the human APPSWE, TauP301L, and PS1M146V genes linked to AD and FTD
3α,5α-THP	3α,5α-tetrahydroprogesterone, allopregnanolone
3α-HSD	3α-hydrosteroid dehydrogenase
3β-HSD	3α-hydroxysteroid dehydrogenase-D5Δ5→Δ4D4 isomerase
3β-HST	3β-hydroxysteroid sulfotransferase
5α-DHP	5α-dihydroprogesterone
17β-HSD	17α-hydroxysteroid dehydrogenase
